# Assessing and addressing vulnerability in pregnancy: General practitioners perceived barriers and facilitators - a qualitative interview study

**DOI:** 10.1186/s12875-022-01708-9

**Published:** 2022-06-03

**Authors:** Louise Brygger Venø, L. Bjørnskov Pedersen, J. Søndergaard, R. K. Ertmann, D. E. Jarbøl

**Affiliations:** 1 Department of Public Health, Research Unit of General Practice, University of Southern Denmark, Odense, Denmark; 2 DaCHE Danish Centre for Health Economics, Department of Public Health, University of Southern Denmark, Odense, Denmark; 3 Department of Public Health, Research Unit of General Practice, University of Copenhagen, Copenhagen, Denmark

**Keywords:** Vulnerability, Pregnancy, Facilitators, Barriers, Antenatal care, General practice, Assessment, Mental health care, Preventive health care, Psycho-social

## Abstract

**Background:**

Vulnerability due to low psychosocial resources increases among women in the fertile age. Undetected vulnerability in pregnancy is a major contributor to inequality in maternal and perinatal health and constitutes a risk of maternal depression, adverse birth outcomes,—i.e. preterm birth, low birth weight, and adverse outcomes in childhood such as attachment disorders. General practitioners (GPs) have a broad understanding of indicators of vulnerability in pregnancy. However, less than 25% of pregnant women with severe vulnerability are identified in Danish general practice. The aim was to explore GPs’ perceived barriers and facilitators for assessing and addressing vulnerability among pregnant women.

**Methods:**

A qualitative study with semi-structured focus group interviews with twenty GPs from urban and rural areas throughout the Region of Southern Denmark. A mixed inductive and deductive analytic strategy was applied, structured according to the Theoretical Domains Framework (TDF).

**Results:**

Five themes emerged covering twelve TDF domains: (I)knowledge and attention, (II)professional confidence, (III)incentives, (IV)working conditions and (V)behavioral regulations. Prominent barriers to assessment were lack of continuity of care and trust in the doctor-patient relation. Other barriers were inattention to indicators of vulnerability, time limits, unavailable information on patients’ social support needs from cross-sectoral collaborators, and lack of reimbursement for the use of extra time. Fear of damaging the doctor-patient relation, ethical dilemmas and time limits were barriers to addressing vulnerability. Facilitators were increased attention on vulnerability, professionalism and a strong and trustful doctor-patient relation. Behavioral regulations ensuring continuity of care and extra time for history taking enabled assessing and addressing vulnerability, especially when a strong doctor-patient relation was absent.

**Conclusions:**

The TDF disclosed several barriers, especially in the absence of a strong doctor-patient relation. A behavior change intervention of restructuring the organization of antenatal care in general practice might reduce the GPs’ barriers to assessing and addressing vulnerability in pregnancy. The findings may serve as a guide for commissioners and policymakers of antenatal care on the GPs’ support needs when providing antenatal care to vulnerable pregnant women.

**Supplementary Information:**

The online version contains supplementary material available at 10.1186/s12875-022-01708-9.

## Background

Vulnerability due to low psychosocial resources, such as mental health problems, seems to increase among fertile women [1], and undetected vulnerability in pregnancy constitutes a risk of complicating pregnancy [2]. However, vulnerability in pregnancy is not unambiguously defined. Previous studies described vulnerability by psychosocial characteristics, such as; lack of social support, living alone, being unemployed, having a low level of education [3–9], adverse childhood experiences, poor socioeconomic status [10], stressful life events during pregnancy [6] and a history of domestic violence or abuse [11]. Additionally, many vulnerable women consume alcohol above the high-risk level [1].

Vulnerability exists in all groups of patients regardless of age and sex. Studies have explored general practitioners (GPs) perceived challenges when assessing vulnerability in general among miscellaneous patient groups, e.g. refugees with mental health problems [12], patients with medically unexplained symptoms [13] and frailty among elderly patients [14, 15]. However, assessing vulnerability in pregnant women may differ in the sense that it is part of preventive health care among women, who by default are healthy. This challenges the GPs in identifying the group of vulnerable women in need of extra support. Furthermore, the topic differs because there are two recipients needing support, the vulnerable pregnant woman and the coming child. Vulnerability in pregnancy is a major contributor to inequality in maternal and perinatal health [16, 17] and constitutes an early risk factor for the debut or relapse of depression during pregnancy or postpartum depression [3, 5, 8, 18]. Among pregnant women, 7–12% are diagnosed with depression [19, 20] and 3–8% are diagnosed with an anxiety disorder [21]. Absence from antenatal care may be the consequence of being vulnerable during pregnancy, as vulnerability may affect the ability of help-seeking [21, 22]. Vulnerability in pregnancy is significantly associated with adverse birth outcomes – i.e. preterm birth, low birth weight and low APGAR scores [23] and adverse outcomes in childhood – i.e. attachment disorders, emotional problems and symptoms of attention deficit hyperactivity disorder [24, 25].

In Denmark, all pregnant women are offered a first early pregnancy consultation with their GP for the purpose of assessing the pregnant women's comorbid risks and psychosocial resources [2]. Danish GPs’ have a broad understanding of indicators of vulnerability in pregnancy [26]. However, less than 25% of pregnant women with severe vulnerability are identified in Danish general practice [27], and only half of the pregnant women with perinatal depression and anxiety are identified in primary care in the UK [28]. Studies have explored GPs’ perceived barriers for assessing perinatal mental health disease(s) [29–31], while barriers for assessing vulnerability, as a broader concept and a predictor of perinatal mental health disease(s) remain unexplored. Indicators of vulnerability are often hidden and vague [26], and GPs’ diagnostic reasoning is often guided by their gut feelings in unclear situations- e.g. pattern recognition from their patient acquaintance, causing senses of alarm indicating that something is wrong despite lacking objective arguments [32–35]. As GPs, our pre-assumptions were formed through years of experience working with antenatal care in general practice and collaborating across sectors on pregnant women. Our pre-assumption was that the GPs have good preconditions for assessing vulnerability due to their knowledge of the patient’s mental health history and ongoing contact with mothers, infants and families. However, we assumed that the GPs are influenced by multiple barriers, such as different attitudes about indicators of vulnerability, fear of stigmatizing pregnant women, as well as insufficient operational conditions in antenatal care. We had experienced how lacking interchange of information on social support needs and substance use treatments between sectors could impede the possibility of assessing vulnerability. The GPs attitudes and understandings of indicators of vulnerability were explored in a separate study [26]. The aim of this study is to explore GPs’ attitudes, experiences and perceived barriers and facilitators for assessing and addressing vulnerability among pregnant women.

## Material and methods

### Design

The study was designed as a cross-sectional qualitative study based on semi-structured interviews with GPs in focus groups. We chose the qualitative methodology to get an in-depth understanding of the GPs various perceived barriers and facilitators in terms of “what, why and how”. The focus group discussions among peers encouraged the GPs to reflect on their own practices and to disclose deficient performances in the area. We invited the GPs to discuss attitudes, experiences, barriers and facilitators in the field without revealing our pre-assumptions. The research group developed the interview guide (Appendix 2), inspired by clinical experience and field observations in social-obstetric outpatient clinics where severely vulnerable pregnant women are treated.

### Setting of general practice and antenatal care in Denmark

Danish GPs are self-employed, working on a contract with the public funder, and the general practice clinics are organized as either single-handed or partnership practices with 2–10 GPs per clinic. The health care system is tax-funded and free of charge for the patient. Most citizens (99%) are registered with a GP, who functions as their gatekeeper to secondary care [36]. Danish antenatal care is a formal collaboration between the GPs, midwives, obstetric departments, and community health visitors [2]. The GP offers three antenatal care visits throughout the pregnancy. During the first pregnancy consultation, the GPs complete the national pregnancy health record concerning, e.g. use of alcohol, smoking and drugs, socio-economic situation, earlier obstetric history and known somatic and psychiatric diseases. The purpose of the first consultation is to support the pregnant woman to obtain optimal individualized conditions for pregnancy and birth with respect to her physical and mental health.

### Research team and reflexivity

The main author is a GP and Ph.D.-student and has attended courses in designing and authoring qualitative articles. The remaining author group are senior researchers with experience in qualitative and quantitative research methodology.

### Selection and recruitment

The study used purposive sampling to include GPs of both sexes with varying levels of experience from urban and rural practices throughout the Region of Southern Denmark (see table in Appendix 1). The GPs were contacted via letter, telephone, e-mails, and snowball sampling. Due to slow recruitment, the included GPs represented only partnership practices. We contacted almost 60 GPs, and their main reason for decline was a high workload.

### Interviews

Focus group interviews were conducted by LBV and DEJ between March 2019 and January 2020. The interviews lasted approximately 60-min and took place at the research unit or in the local area of the respondents. Twenty GPs participated in five qualitative focus groups with 3–6 GPs in each group. The first interview was a pilot, with GPs (*n* = 5) working as part-time researchers in our research unit and therefore had prior knowledge of the research team. The interview guide provided a flexible frame with open-ended questions about GPs’ attitudes, experiences, perceived barriers and facilitators while welcoming clinical examples. Ongoing adjustments of the interview guide were made to elaborate on new perceptions. Sampling ceased when no new barriers and facilitators emerged. All interviews were audio-recorded and transcribed verbatim by LBV and uploaded to NVivo. To increase transparency, the study followed the Consolidated criteria for Reporting of Qualitative research checklist (COREQ) [37] (Appendix 3).

### Theoretical frame

Initially, we applied a pragmatic clinical approach to explore the research question concerning the GPs’ attitudes, experiences, barriers and facilitators. During the interview process, we searched for theories to support the data interpretation. We found the Theoretical Domains Framework (TDF) useful as a theoretical lens to understand the GPs’ behavior of assessing and addressing vulnerability. The TDF-domains cover a person’s capability, opportunity and motivation (including attitudes and beliefs), and it enables us to view the cognitive, affective, social and environmental influences on behavior [38, 39].

The TDF is based on theories of human behavior, and it was developed to understand the context of behavior – i.e. identify existing barriers and facilitators and to promote understanding of how to change health professional behavior [38, 40, 41]. It is derived from an integration of 33 theories and 128 constructs from behavioral theory, resulting in 14 theoretical domains useful for categorizing barriers and facilitators to specific behaviors [38, 40, 41]. TDF has been validated to facilitate research into implementation problems [42], and it has been used in empirical studies exploring the barriers of GPs and other health care professionals when implementing interventions in clinical areas—such as preconception care [43], weight management and smoking cessation during pregnancy [44–46] and the delivery of healthy kids check [47]. A codebook was developed accommodating the TDF domains for the concept of assessing and addressing vulnerability (See Appendix 3). However, acknowledging that the deductive nature of the TDF does not sufficiently cover all aspects of the research questions, it was combined with inductive analysis to ensure the coverage of individual attitudes and experiences.

### Data analyses

The transcripts from the first two focus groups were read thoroughly by all authors before being coded. As illustrated in Table 1, the analysis transitioned between; open inductive coding and deductive thematic coding to domains of the TDF [42]. The inductive coding was inspired by systematic text condensation, which is a pragmatic approach inspired by phenomenology [48]. It ensured an in-depth investigation of themes and subthemes, where the perceived attitudes, experiences, barriers, and facilitators could freely emerge. Additional deductive coding to the domains of the TDF [42] ensured a comprehensive coverage of barriers and facilitators related to behavioral theories. Finally, the inductive and deductive findings were gathered into the identified themes.

**Table 1:**
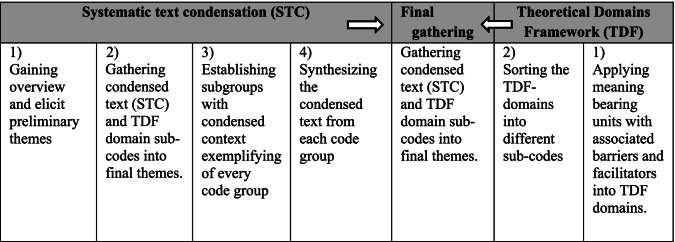
The steps and content of systematic text condensation and TDF

Two authors (LBV and RE) conducted the inductive coding, whereas a single author (LBV) conducted the deductive coding, assisted by JVL. Almost all codes occurred in every focus group interview, and the results were discussed among all authors.

**Fig. 1:**
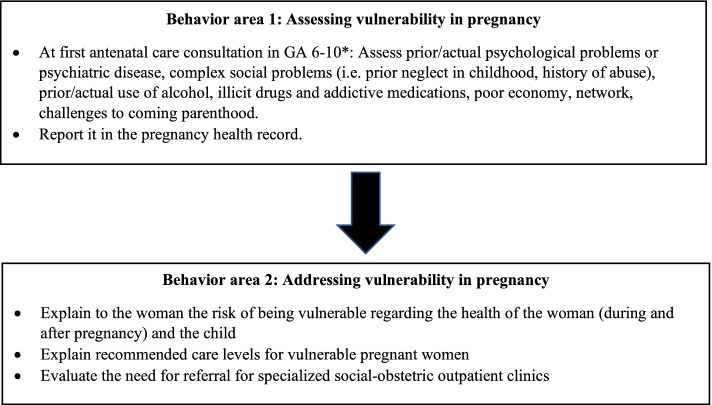
Behavior areas assessing and addressing of vulnerability in pregnancy in general practice (GA = gestational age, * = or pregnancy planning consultations)

In accordance with the Danish guideline recommendations for antenatal care [2], two behavior areas were formulated to guide the analysis (Fig. 1). The behavior areas were developed by grouping the guideline recommendations for assessing and addressing vulnerability in pregnancy and identifying how these behaviors would be carried out during antenatal consultations. The result section is structured around these behavioral areas.

#### Results

The GPs reported assessing vulnerability among pregnant women based on their familiarity with both the patient and their medical records, in addition to relying on their clinical experience and intuition. From the inductive and deductive analysis, five main themes emerged concerning barriers and facilitators for assessing and addressing the issue of vulnerability in pregnancy: (I)*knowledge and attention,* (II)*professional confidence*, (III)*incentives*, (IV) *working conditions* and (V)*behavioral regulations*. The underlying TDF domains according to the themes, and their relation to behavior areas (assessing and addressing) are shown in Table 2. Where no citations are given, the text refers to condensed text according to the inductive analysis. For a detailed overview of the content of the TDF domains, see the codebook in Appendix 4. The data material did not contain meaning units of all TDF domains, which explains the two empty categories in Table 2.

**Table 2:** GPs perceived barriers and facilitators for assessing and addressing vulnerability, according to TDF-domains

**Behavioral domains**	**Behavior area 1: Assessing vulnerability in pregnancy**		**Behavior area 2: Addressing vulnerability in pregnancy**	
TDF domains	Barriers	Facilitators	Barriers	Facilitators
***Theme I: Knowledge and attention***				
*Knowledge*	Uncertainty of levels of antenatal care		Uncertainty of collaborative opportunities	
*Skills*	Lacking training in how to manage vulnerable pregnant women Losing overview in the pregnancy record	Coping skills, assessing vulnerability guided by the pregnancy record		Communication skills, being honest and trustworthy
*Memory, attention and decision processes*	Inattention to vulnerability due to the patient’s normal visual appearance	Attention to the patient’s social life and living conditions		
***Theme II: Professional confidence***				
*Social/professional role and identity*		Judged a meaningful task for GPs	Ethical dilemmas, balancing the needs of the patient versus the needs of the coming child	Keeping professional obligations in mind Sharing personal experience and attitude
*Beliefs about capability*	Absence of doctor-patient relation, No trust in gut-feeling Poor confidence in assessing vague indicators of vulnerability	Existing strong doctor- patient relation Professional confidence, trusting their gut feeling		Durable patient-alliance from existing strong doctor-patient relation
*Beliefs about consequences*			Fear of breaking the patient-alliance	
*Optimism*	No coding			
*Intentions*	Blind to problems due to long standing relations, not asking			
*Goals*	No coding			
*Emotion*	Empathy and trust from longstanding relation		Having sympathy Feeling sorry for the patient	
***Theme III: Incentives***				
*Reinforcement*	Lacking economic compensation for the use of extra time	Desired changes to the collective agreement		Desired changes to the collective agreement
***Theme IV: Working conditions***				
*Environmental context and resources*	Missing information in medical records, Time constraints Lacking continuity Delegating ANC to staff/GP trainees No home visits for vulnerable families Patient conditions		Time constraints limit proper sensitive addressing of vulnerability	
*Social influences*		Influences from relatives and colleagues who know the patient		
***Theme V: behavioral regulations***				
*Behavioral regulations*		Local prompts and structure changes facilitating vulnerability assessment		Local structure changes ensuring time for proper addressing of vulnerability

The TDF domains are shown in left column in italics and categorized in themes (I-V). Empty boxes refer that no barriers/facilitators were found in the data material

##### Behavior area 1: Assessing vulnerability in pregnancy

### Theme I: Knowledge and attention

Assessing vulnerability in pregnancy consists of several tasks: having clinical *‘*knowledge’ of indicators of vulnerability, having procedural ‘knowledge’ of the antenatal care pathways for vulnerable women, having the interpersonal ‘skills’ to assess vulnerability and the ‘memory and attention’ to focus on vulnerability.

Some GPs reported that assessing vulnerability was facilitated by the ‘skills’ of being guided by the pregnancy record when exploring the women’s medical history, combined with having ‘memory, attention and curiosity about the women’s living conditions and social lives.

“*Some things you’ll automatically assess when going through the pregnancy record – when you’re asking about alcohol, medication, abuse and smoking. But the thing with knowledge about the family [indicators of family vulnerability due to social and psychological problems], you can’t record this in the pregnancy record if you don’t know the family, are not asking them or are having the sensation that this family is vulnerable.”* (female GP, > 45 years)

Lack of procedural ‘knowledge’ was a barrier when assessing vulnerable pregnant women, i.e. being uncertain about the content of the different levels of antenatal care.

*“I have a large group [of pregnant women] that I would call fragile, where I’m in doubt if I should start up something– the anxious ones, where I’m thinking they don’t have the right resources as parents. This is a group we see very often, and who’s difficult to allocate to the right supportive care(..) I’m often confused about the content of the different levels in antenatal care - are there any other options for her?”* (female GP, > 45 years)

Some GPs reported barriers, such as lacking ‘skills’ due to receiving inadequate training in handling vulnerable pregnant women and lack of ‘attention’ to indicators of vulnerability in pregnancy.

*“There are so many areas you have to address when filling out the pregnancy record, so your focus might only be in filling it out correctly, thereby missing some of the details about vulnerability”* (female GP, < 45 years)

Furthermore, GPs reported lack of ‘attention’ as a barrier to assessing vulnerability when perceiving a pregnant woman as having normal resources due to her normal physical appearance. One GP was surprised when she diagnosed one of her upper-class female patients in late pregnancy with depression. Moreover, some GPs reported missing histories of abuse, because the women presented with normal physical appearances indicating strong resources.

### Theme II: Professional self-confidence

The GPs agreed that assessing vulnerability in pregnancy was part of their ‘social and professional role and identity*’*. GPs with many years of experience seemed to have professional confidence and trust in their own gut feelings, which made them ‘believe in their capability’ to assess vulnerability.

*“It’s your impression when they enter, and you see them, listen to them and sense them – then you’re almost ready to evaluate whether they are vulnerable. It’s intuition, knowledge and experience. We almost know it at once, this is not good… here’s somebody who needs extra care”* (female GP> 45 years).

Having a strong doctor-patient relation was a great facilitator, increasing the GPs’ ‘beliefs in their capabilities*’* assessing vulnerability, due to the familiarity with the woman’s chronic diseases and prior psychological and family history.

Some GPs expressed poor ‘beliefs in their capabilities’ assessing the vague indicators of vulnerability, when their perception of a woman’s vulnerability was only based upon an impression. Especially GPs with fewer years of work experience expressed poor ‘beliefs in their capabilities*’* of judging the most important factors when going through the pregnancy health record, because of the complex task of differentiating the vast amount of information.

“*When filling out the pregnancy record, there is some basic information (…), and I tend to take the low-hanging fruits spending time on the basic stuff, where I lose focus on the important things”* (female GP < 45 years).

A longstanding strong doctor-patient relation was also a possible barrier to assessing vulnerability, e.g. underestimating the possible vulnerability, not making a point of asking about alcohol or drug consumption when not presuming it as a problem. This was coded into the domain ‘intentions’. Furthermore, ‘emotions’ due to empathy or longstanding trust could be a barrier; as a GP said:

*“Sometimes I think we underestimate when we’re having such a basic trust and positive attitude towards our patients, so it might just appear to us quite late, that the problem is bigger than expected”* (male GP, > 45 years)

### Theme III: Incentives

Some GPs reported that one aspect of the current collective agreement for antenatal care could be a barrier. Currently, no economic reimbursement is given to compensate for the use of longer consultations when assessing vulnerability in complex cases, thereby giving a lesser degree of ‘reinforcement’.

*“Throughout my years as a GP, I clearly feel how the quick consultations are becoming fewer and the number of complicated consultations is increasing. I typically see the same number of patients now as I did 25 years ago, but it takes me at least one to two hours longer every day. Though it’s a general problem - time has run away regarding the size of remuneration per consultation slot. If we’re giving double consultation slots, it is not being remunerated.”* (male GP > 45 years)

Some GPs perceived that potential changes to the collective agreement could facilitate assessing vulnerability, i.e. allowing prolonged time used on antenatal care to be reimbursed.

### Theme IV: Working conditions

GPs reported several barriers to assessing vulnerability related to their ‘environmental context and resources’*.* The lack of available information on many indicators of vulnerability in the women’s medical records was a barrier. This could be diagnoses of personality disorders or information on social support provided by the authorities, indicating lacking resources in the woman or her family. Furthermore, the often-delayed transfer of new patients’ medical records was also perceived as a barrier to assessing vulnerability.

*“I saw a pregnant woman, 35 years old, who was an educated nurse - but not working, and when I asked why - she told me she had a personality disorder, which is not a disease and therefore not registered in her file, and she was not taking medication for it…. That worried me”* (female GP > 45 years)

Other barriers were organizational constraints such as a busy working environment, often being behind schedule, and limited time dedicated to antenatal care consultations. These barriers limited proper history taking, especially when a pre-existing doctor-patient relation was absent.

Similarly, lack of continuity when shifting doctors consult the patients were perceived as a barrier to assessing vulnerability. Some GPs reported feeling a loss of control over the assessment process when delegating pregnancy consultations to younger GP trainees.

*“We had a case with a young pregnant woman with a hearing disability and many psychiatric challenges. By coincidence, I saw her pregnancy record in the reception. One of our GP trainees had filled out her record, and had just concluded that she had no need for extra care – which was certainly not what I would have concluded/recommend”* (female GP < 45 years)

The GPs expressed diverse attitudes regarding the consequences of delegating antenatal care consultations to staff members. Some GPs perceived that delegating antenatal care would be a barrier to properly assessing vulnerability due to the staff’s lack of knowledge of the family and lack of experience in navigating the medical system.

The fact that home visits for deprived families are not prioritized due to time constraints were reported as a barrier, due to the risk of missing important details about the family’s resources.

At last, the GPs related barriers to assessing vulnerability due to conditions of the pregnant women, which were out of their control. Some women were reluctant to disclose vulnerability due to previous negative experiences with social authorities. Assessing vulnerability among pregnant women with migration background were perceived as a special challenge due to their poor understanding of the language, culture and the health system- including the meaning of the prophylactic antenatal care visits. Furthermore, a barrier was the general poor socio-economy among these women limiting the use of proper translator assistance.

The concept of being a “family physician”, getting to know entire generations of families, was perceived to facilitate the assessing of vulnerability. A young GP described how ‘social influences’ from experienced GP colleagues or relatives helped her point out women with complicated family histories, and understand that she needs extra support during pregnancy.

*“My group of patients are quite old. (..), and many of their kids are now having children as well. In that way, my elderly GP colleague knows a lot about family relationships, which is my benefit”* (female GP < 45 years)

### Theme V: Behavioral regulations

As self-employed, some GPs had made ‘behavioral regulations’ into the organizational structure of antenatal care in their clinic to facilitate assessment, i.e. prioritizing extra consultations for history taking and relation gaining when no doctor-patient relation existed. Furthermore, the following initiatives were perceived to promote assessing vulnerability in pregnancy: planning early consultations immediately after confirmed pregnancy to identify severe psychosocial risks and ensuring continuity among GPs by marking the medical files of the women known to be vulnerable. Some GPs changed structural habits by requiring a senior GP who knows the patient to approve the GP trainees’ antenatal care decisions.

### Behavior area 2: Addressing vulnerability in pregnancy

#### Theme I: Knowledge and attention

Insufficient procedural *‘*knowledge’ of options for collaboration was perceived as a barrier for addressing vulnerability, e.g. uncertainty regarding collaborative opportunities with community health visitors and referral to social-obstetric support.

“*I saw a pregnant woman where I was worried. A well-educated woman with a history of personality disorder. I had this sensation that she did not have the competence to be a parent, but I couldn’t give her a diagnosis, I couldn’t send her to the social-obstetrics clinic (...) I had this deep concern that I didn’t know how to handle (…) because I couldn’t say to her, ‘I’m in doubt that you can handle this [being a mother]’”.* (female GP, >45 years)

An important facilitator for addressing vulnerability was having good communication ‘skills’ by being trustworthy and honest with the woman when addressing the need for social-obstetric and municipal support.

*“It’s about presenting it as an offer (…), so you are selling it and really mean it. When you express doubt about their parenting skills and talk about forcible child removals, the conversation shifts character. As long as you believe it will succeed, then you can sell it, but you have to be honest because it’s about trust (…) it’s a terrible setback if you break their trust*” (male GP, < 45 years)

#### Theme II: Professional self-confidence

Some GPs reported barriers regarding ethical dilemmas related to their ‘social/professional role and identity’*,* when perceiving a pregnant woman being vulnerable to such a degree, that they doubted her parenting skills.

“*It can be hard to address if a vulnerable woman insists on carrying through with a pregnancy. As a doctor, you are sometimes in a severe dilemma. On the one hand, you have to think of the coming child, who might be best served by being removed at birth, and on the other hand, you have your patient that you have to help and take care of their interests without pushing them away”* (male GP > 45 years)

A GP described how sharing her personal experiences and perspectives with a woman enabled her to sensitively address concerns of vulnerability.

“*There, I simply jumped out of my professional role and said that "my own experience of becoming a mother is that it actually requires a certain energy - and there are situations where you do not know yourself” .. so that one becomes completely universally human.[…] I’m worried about whether you could be lonely in the process of becoming a mother because you are sitting there all alone without social relations*” (female GP> 45 years old)

Low professional confidence was a barrier in addressing vulnerability, due to the GPs’ ‘beliefs about consequences’ of breaking their patient-alliance. Furthermore, GPs feared stigmatizing the patient leading her to seek a new GP.

*“I need to have a strong suspicion before I raise the topic of vulnerability with her, or else we’ll get a very bad communication, and she will probably change to another GP, if I say something at all about vulnerability”*(female GP, > 45 years)

Additionally, the GP perceived ‘emotions’ for the patient due to their longstanding relation were reported as a possible barrier.

*“I have patients I have known for very long, with whom I have a good relationship(…) then they get pregnant which was not planned, and I think, how can they manage being a mother of a child(…), then all of a sudden I am feeling sorry for the patient, and maybe I end up doing nothing – because I know the family so well”* (male GP > 45 years)

Keeping their ‘professional role’ in mind was a facilitator for the GPs when addressing vulnerability. Some GPs suppressed their ambition of being the likeable doctor, and held on to their decision of addressing the need for referral to social-obstetric support, despite reluctance among the woman or her partner.

Also, having a strong doctor-patient relation with a durable alliance was perceived as a facilitator, as it increased the *GPs’ ‘*beliefs in their capability ‘ of addressing vulnerability from their knowledge of the woman’s personality.

#### Theme III: The incentives

GPs reported that *‘*reinforcements’, changing the collective agreement to include remuneration for extra time spent on challenging antenatal consultations of vulnerable pregnant women, were perceived to motivate addressing vulnerability and the need for extra care.

#### Theme IV: Working conditions

‘Environmental context and resources’ including shortness of time available for communicating concerns sensitively were perceived as a barrier for addressing vulnerability.

*“It is also about having the time to present it – not just “oh, so you’re having some psychosocial problems – but now I have to move on to the next point” – so if you ask about these things, you need the time to address them properly”* (female GP > 45 years)

#### Theme V: Behavioral regulations

‘Behavioral regulations’ facilitating the addressing of vulnerability were local structure changes, such as prioritizing extra consultation for pregnant women without an existing doctor-patient relation, and marking the files of women with known vulnerability to ensure continuity in her care.

## Discussion

### Statements of principal findings

Barriers and facilitators for assessing and addressing vulnerable pregnant women were represented in almost all TDF domains relating to the themes: knowledge and attention, professional confidence, incentives and organizational working conditions. The theme behavioral regulation featured the GPs applied strategies to relieve the barriers. Remarkably, lack of attention, misjudging the resources of pregnant women from her appearance, could delay the assessment of vulnerability. The absence of a strong-doctor patient relation made the GPs in-confident in assessing vulnerability, whereas a strong doctor-patient relation facilitated the assessing and addressing of vulnerability. Interestingly a strong doctor-patient relation could also limit the GPs from addressing vulnerability due to emotions and fear of breaking the patient-relation. Ethical dilemmas challenged the GPs’ professionality when addressing vulnerability. Organizational working conditions-i.e. lacking information in medical records, time limits s and lack of continuity were barriers to assessing and addressing vulnerability. Behavioral regulations facilitated the assessing and addressing of vulnerability through ensuring continuity and prompting extra time for history taking and relation gaining when a doctor-patient relation was absent.

### Strengths and weaknesses of the study

A strength of this study was the semi-structured qualitative approach using focus group interviews. The flexible interview guide encouraged free discussion, with an intense dialogue between participants and provided a deeper insight into the GPs’ perceptions of the subject. Ongoing adjustments to the interview guide ensured coverage of all emerged perceptions of barriers and facilitators. Another strength is the high information power [49] due to continuing interviews until reaching a study sample large enough to answer the research question, thereby increasing credibility. The use of the COREQ criteria ensured transparency [37]. Furthermore, the combined use of open inductive and focused deductive coding was a strength [42, 48]. TDF has been validated to facilitate research into implementation problems [42], and thus it was deemed both an appropriate theoretical foundation and appropriate model for exploring the study aim. The deductive nature of the TDF may have limited our interpretation of the data. However, we accommodated this with the mixed inductive and deductive coding. The inductive coding ensured an in-depth investigation where themes and sub-themes emerged freely, and the application of TDF as a theoretical frame gave a comprehensive coverage of a multitude of barriers and facilitators of the GPs’ behavior when assessing and addressing vulnerability.

Since the research group possessed years of experience working with antenatal care in general practice and collaborating with antenatal care partners, this gave a comprehensible insight into the working conditions and possible challenges for the GPs when assessing and addressing vulnerable pregnant women. However, we acknowledged that the generation and interpretation of data might have been affected by our prior experience, and that other professional expertise might have challenged our pre-assumption.

This study sought to achieve a purposeful sample of GPs but ended with a convenience sample due to slow recruitment. Due to the slow recruitment, we accepted small-sized focus group interviews with 3–6 participants, which may have limited the broadness of the discussions. However, a small group may also have ensured confidentiality among peers and made it easier for the respondents to make themselves heard. Only GPs from partnership practices participated, and these participants might have been the ones naturally interested in the topic, thereby limiting the transferability of the findings. However, diversity was achieved, as participants were roughly distributed by sex, seniority and practice location from municipalities of different socioeconomic layers. Moreover, the GPs in the study represented a multitude of barriers and facilitators for assessing and addressing vulnerable pregnant women.

### Findings in relation to other studies

The result from this study supports the findings of studies from comparative countries -i.e. the UK, Ireland and Australia, exploring GPs’ perceived barriers to assessing perinatal mental health problems [21, 28–31, 50, 51]. These studies confirm barriers, such as lack of knowledge of collaboration opportunities in the antenatal care system, lack of knowledge of the consequences of perinatal mental health problems, and lack of attention to assessing perinatal mental health problems [21, 29, 30, 50, 51]. The GPs lacked the skills and confidence to identify and manage perinatal mental health problems due to variable levels of training [28, 30], and similar environmental barriers were shown, such as; time limits, language barriers and lack of guidelines [21, 29, 30, 50, 51]. This study covers a broader perspective, since the concept of vulnerability includes more than perinatal mental health – e.g. social factors and somatic diseases [9, 26]. To our knowledge, this study is the first to apply behavioral change theory with the TDF to analyze GPs’ barriers and facilitators for assessing and addressing vulnerability in pregnancy Since the purpose of the TDF is to identify behavioral domains that warrant further investigation [39, 42, 52], findings from this study can possibly facilitate the generation of future intervention strategies optimizing antenatal care, such as recommended behavioral changes for assessing vulnerable pregnant women in general practice. Recently, scientists of behavior change has developed a web-based tool build on evidence linking behavioral domains to possible mechanism of actions [53–55].

A consistent facilitator, was the importance of having a strong trusting doctor-patient relationship with the women; thus, increasing the GPs’ confidence and perceived competence, thereby supporting the value of the patient list system [36]. This was confirmed in studies of GPs’ assessing of perinatal mental health problems [30, 31]. Likewise, women prefer disclosing negative experiences and emotions in the context of a continuous relationship with a professional [56]. Contrarily, this study also found that a strong doctor-patient relation might be a barrier for the GPs’ when addressing vulnerability, due to concerns of damaging the doctor-patient relation or due to emotions of empathy and trust. This illustrates how GPs’ professionalism can be challenged by their dual role, balancing the interests of both the woman, the coming child and the health care authorities. Professionalism also reflects employing judgement and coping with uncertainty [57], which can be challenging in cases where vulnerability can only be detected by the GPs impression [26]. Despite this, GPs were aware of the importance of maintaining their professional role when addressing vulnerability. Afterall, trust is not linked to an eager-to-please attitude. Moreover, realistic and trustworthy medicine implies that the GPs must address vulnerability, even though it can challenge the alliance with the patient [58].

Other studies supported the findings regarding organizational barriers such as time limits and lack of continuity, as barriers for assessing perinatal mental health [28, 30, 51]. Along those lines, the Royal College of General Practitioners in the UK recently recommended that “politicians should take action to reduce pressure on general practice to enable longer consultations to be offered to women at risk of or with perinatal mental health problems” [28].

The findings of this study is supported by the concept of street level bureaucracy by Lipsky [59]. The GPs “*must use their personal discretion to become inventive strategists, by developing ways of working to resolve excessive workload, complex cases and ambiguous performance targets*”. Vulnerability as a concept is ambiguously defined, and the GPs must prioritize their working effort in between primary prevention and secondary prevention among patients with multimorbidity.

Organizational structural regulations were suggested to facilitate assessing and addressing of vulnerable pregnant women, especially in the absence of a pre-existing doctor-patient relation. Suggestions were ensuring doctor-patient continuity, sharing knowledge with colleagues about female patients known to be vulnerable, and prioritizing extra time to uncover vulnerability in pregnant women. This is in line with recommendations from the recently updated NICE guideline in the UK regarding assessment of antenatal mental health [16].

### Meaning and implications of the study

In conclusion, this study showed the contrasting influences on the doctor-patient relation. On the one hand a strong and trusting doctor–patient-relation was perceived to ease the assessing and addressing vulnerability. However, it could also limit the addressing of vulnerability due to the GPs’ emotions for the patient, and fear of breaking the doctor-patient relation. In the absence of a pre-existing doctor-patient relation, then precautions are needed to ensure proper assessing of vulnerability. In their daily work GPs may consider discussing suspicions of vulnerability in pregnant women with colleagues who know the woman, dedicating extra time and prioritizing continuity.

Based on this study’s observed barriers, different behavior changing interventions might be needed, e.g. continuous medical education focusing on increasing the GPs confidence in assessing and addressing vulnerability among pregnant women. Additionally, a minor scale intervention study might be relevant – i.e. in a selected region testing whether available increased remuneration for additional time to assess vulnerability could reverse the environmental barriers of time constraints. Future studies should aim to explore the proportion of barriers among a larger group of GPs, and explore the association with the organization of antenatal care, GP, and practice characteristics. Moreover, GPs perceived challenges in relation to cross-sectoral collaboration associated with antenatal care for vulnerable pregnant women needs attention. The findings from this study and future studies, may serve as a guide for commissioners and policy makers of antenatal care services, who may consider accommodating and overcoming the observed barriers – i.e. changing some of the factors related to the environmental context and resources.

## Supplementary Information

**Additional file 1.** **Appendix 1.** Table 1: Participant demographic details

**Additional file 2.** **Appendix 2.** Interview guide

**Additional file 3. Appendix 3.** COREQ items

**Additional file 4. Appendix 4.** Codebook 

## Data Availability

The dataset generated and analyzed during the current study are not publicly available, due to them containing information that could compromise research participant consent, but are available from the corresponding author on reasonable request. Data supporting the findings of this study was used under a license granted specifically for the current study, and therefore is not publicly available according to the data protection regulations from the Danish Data Protection Agency.
