# Supplementary material for: Assessing and addressing vulnerability in pregnancy: General practitioners perceived barriers and facilitators - a qualitative interview study
Source: BMC Prim Care. 2022 Jun 3;23:142. doi: 10.1186/s12875-022-01708-9 (PMC9164392; doi:10.1186/s12875-022-01708-9)
Supplement: Supplementary file 1 — Additional file 1. Appendix 1. Table 1: Participant demographic details [file 12875_2022_1708_MOESM1_ESM.pdf]

Appendix 1

Table 1: Participant demographic details

Years of experience	Practice type	Practice area	Gender
0 years (GP trainees) (3)<br>1-5 years (5)<br>6-10 years (2)<br>11-15 years (5)<br>> 15 years (5)	Single-handed practices (0)<br>Partnership practices (20)	Urban area (5)<br>Semi-urban area (11)<br>Rural area (4)	Female (12)<br>Male (8)
