# Supplementary material for: Assessing and addressing vulnerability in pregnancy: General practitioners perceived barriers and facilitators - a qualitative interview study
Source: BMC Prim Care. 2022 Jun 3;23:142. doi: 10.1186/s12875-022-01708-9 (PMC9164392; doi:10.1186/s12875-022-01708-9)
Supplement: Supplementary file 3 — Additional file 3. Appendix 3. COREQ items [file 12875_2022_1708_MOESM3_ESM.pdf]

COREQ items

Domain 1: Research team and reflexivity

Personal Characteristics

1. Interviewer/facilitator: The first author LBV conducted the interviews with DEJ as an experienced moderator
2. Credentials: The main author LBV is a GP and Ph.D. student at the Research Unit of General Medicine at the University of Southern Denmark. DEJ and JS are part-time GPs and professors at the Research Unit of General Medicine at the University of Southern Denmark. RE is a part-time GP and associate professor at the Research Unit of general medicine, University of Copenhagen. LIB is a health economist and associate professor at the Research Unit of General Medicine at the University of Southern Denmark.
3. Occupation: LBV work as a part-time GP and part-time Ph.D. student
4. Gender: LBV is female
5. Experience and training: LBV have attended Ph.D. courses in qualitative study designs and writing qualitative articles. DEJ, JS and RE are all senior researchers with experience in qualitative and quantitative research traditions. LIB is a senior researcher with expertise in survey studies

Relationship with participants

6. Relationship established: The first interview was a pilot with GPs (n=5) working as part-time researchers in our research unit and therefore had prior knowledge of the researcher team.
7. Participant knowledge of the interviewer: Prior to the interviews, the interviewer presented the study aim but without giving our prior experiences and pre-assumptions in the field
8. Interviewer characteristics: The interviewer acted friendly and like-minded among participants. The interview guide provided a flexible frame with open-ended questions about the GPs' perceived barriers when collaborating and reporting on vulnerability among pregnant women and welcoming clinical examples. Ongoing adjustments to the interview guide were made to elaborate on new perceptions.

Domain 2: study design

Theoretical framework

9. Methodological orientation and theory: We chose the qualitative methodology to explore GPs perceived barriers and facilitators when collaborating in ANC and reporting on vulnerability in pregnant women. A qualitative design enabled us to explore the GPs perceived barriers and facilitators in terms of "what, why and how". The safe environment during the interview encouraged them to disclose situations of deficient performance when collaborating or reporting on vulnerable pregnant women, and the dialogue rendered the GPs to reflect on their practices.
   We applied a pragmatic clinical empirical approach not driven by a prior established theoretical framework. However, recognizing that our stance is always affected by theory, we searched for theories to support our data interpretation during analysis. We chose the Theoretical Domains Framework (TDF)(1) as a theoretical model to understand the GPs' behavior and perceived barriers and facilitators when collaborating and reporting on vulnerable pregnant women.

Participant selection

10. Sampling: The study aimed to recruit a purposive sample of GPs concerning; gender, years of experience, practice type and various practice areas throughout the Region of Southern Denmark, representing communities of all socio-economic layers.
11. Method of approach: Respondents were recruited via letter, telephone, e-mail, and snowball sampling.
12. Sample size: Due to slow recruitment, the end sample consisted of a convenience sample of twenty GPs representing only partnership practices
13. Non-participation: Almost 60 GPs were contacted, and the main reason for declining was a high workload.

Setting

14. Setting of data collection: The interviews lasted approximately 60 minutes and took place at the research unit of general practice in Odense or in the local practice area of participating GPs.

15. Presence of non-participants: No other besides participants, the interviewer LBV, and moderator DEJ was present
16. Description of sample: A heterogeneous sample of GPs with respect to gender (12 females, 8 males), experience with both GP trainees (n= 3), 1-10 years of experience (n=7), 11 years and above experience (n=10), from urban (n=5), semi-urban (n=11) and rural areas (n=4).

Data collection

17. Interview guide: The interview guide provided a flexible frame with open-ended questions about the GPs' perceptions of enablers and barriers in collaboration and reporting on vulnerable pregnant women, and welcoming clinical examples. Ongoing adjustments to the interview guide were made to elaborate on new perceptions.
18. Repeat interviews: five focus group discussions were made, which included the pilot test
19. Audio/visual recording: All interviews were audio-recorded and transcribed verbatim by LBV
20. Field notes: the author made field notes after almost all interviews about the characteristics of the interview
21. Duration: each interview lasted approximately 60 minutes
22. Data saturation: data saturation was discussed among authors
23. Transcripts returned: No transcripts were returned to participants for comments or corrections

Domain 3: analysis and findings

Data analysis:

24. Number of data coders: All authors read the first two interviews. After the authors discussed the themes, LBV and RE conducted the initial inductive coding according to systematic text condensation, which is a pragmatic approach inspired by phenomenology(2). During the process of inductive analysis, we chose to apply TDF as a theoretical model to guide the deductive analysis. LBV conducted the deductive coding to TDF domains and sub-coding, assisted by DEJ and JVL. JVL is an MD, PhD with experience in behavioral research. The research team discussed and reflected on the findings until a consensus was reached.
25. Description of the coding tree: The combined inductive-deductive coding is illustrated in table 1, and the result of the deductive coding is shown in table 2.
26. Derivation of themes: The themes were identified from the data in both the inductive and deductive analysis.
27. Software: NVivo pro version 12 was used to organize the data
28. Participant checking: As no transcripts were returned to participants, no participants provided feedback.

Reporting

29. Quotations presented: quotations were presented to illustrate themes identified by participant number.
30. Data and findings consistent: Consistency was found between the data and the findings, as the findings were recontextualized against the original interview material, which is one of the steps in the Systematic Text Condensation.
31. Clarity of major themes: The major themes covering twelve TDF domains were clearly presented in the findings.
32. Clarity of minor themes: categories of minor themes were presented in the text and table 2.

References

1. Cane J, O'Connor D, Michie S. Validation of the theoretical domains framework for use in behaviour change and implementation research. Implement Sci. 2012;7:37.
2. Malterud K. Systematic text condensation: a strategy for qualitative analysis. Scand J Public Health. 2012;40(8):795-805.
