# Supplementary material for: Assessing and addressing vulnerability in pregnancy: General practitioners perceived barriers and facilitators - a qualitative interview study
Source: BMC Prim Care. 2022 Jun 3;23:142. doi: 10.1186/s12875-022-01708-9 (PMC9164392; doi:10.1186/s12875-022-01708-9)
Supplement: Supplementary file 4 — Additional file 4. Appendix 4. Codebook [file 12875_2022_1708_MOESM4_ESM.pdf]

Appendix: Theoretical Domains Framework (TDF), domains and constructs coding manual

Adapted from Michie et al 2005 and Cane et al 2012

	**Domains and belonging constructs**<br>The domains are registered by number<br>Italic text refers to constructs adapted from Michie 2005 and Cane 2012	**Examples from the relevant data**
COM-B: psychological CAPABILITY	**Knowledge (1)**<br>• Knowledge of condition/scientific rationale<br>• Procedural knowledge (how to do it)<br>• Schemas + mindsets + illness representations	• Statements about having/not having /wanting **factual knowledge** of indicators of vulnerability and the following risks in pregnancy<br>• Statements about having/not having /wanting **procedural knowledge** of when and how to assess for vulnerability in pregnancy<br>• Statements about having/not having /wanting **scientific rationale** of assessing for vulnerability in pregnancy<br>In the context of this study, knowledge of the conditions/scientific rationale could relate to knowledge about vulnerability indicators, how it can affect the pregnancy, and the risk for the child. Procedural knowledge could relate to knowledge of care pathways in different levels of antenatal care for vulnerable pregnant women. May be both correct and incorrect knowledge.
COM-B: physical and psychological CAPABILITY	**Skills (2)**<br>• Skills Development *(the gradual advancement through progressive stages of an ability or proficiency acquired through training and practice)*<br>• Competence (*one's repertoire of skills, and ability especially as it is applied to a task or set of tasks)*<br>• Ability *(competence or capacity to perform a physical or mental act. Ability may be either unlearned or acquired by education and practice)*	• Statements describing techniques/capability/skills used/how they assess and address vulnerability, and what they write in the pregnancy record<br>• Statements of wanting to develop/improve skills in assessing and addressing vulnerability in pregnancy.<br>In the context of this study, skills may be interpersonal skills (e.g. using empathy, sensitivity, practical advice, promote benefits, non-judgmental approach, terminology, normalization, communication skills etc.). May also be skills adopted to cope in the absence of specific skills training related to assessment of vulnerability.

	• Interpersonal skills *(an aptitude enabling a person to carry on effective relationships with others, such as an ability to cooperate, to assume appropriate social responsibilities or to exhibit adequate flexibility)*<br>• Practice *(repetition of an act, behavior or series of activities, of the to improve performance or acquire a skill)*<br>• *Skill assessment (a judgement of the quality, worth, importance, level or value of an ability or proficiency acquired through training and practice)*<br>• Coping strategies	
COM-B: psychological CAPABILITY	**Memory, attention and decision processes (10)**<br>• Memory: (*ability to retain information or representation of a past experience based on the mental processes of learning)*<br>• Attention: *(awareness, senses are focused selectively on aspects of the environment, readiness to respond to stimuli)*<br>• Attention control: *(concentration on relevant cues and ignore irrelevant cues in a given situation)*<br>• Decision making*: (cognitive process of choosing between two or more alternatives)*	• Statements relating to time/situations etc. when the GPs would remember/forget to assess and address vulnerability in pregnancy.<br>• Statements relating to relying on cognitive approaches to assess and address vulnerability/make a quick decision<br>• Statements relating to cognitive limitations such as forgetting/overseeing/not being able to make the decision<br>In the context of this study, memory, attention and decision processes may relate to the GPs ability to remember to assess and address vulnerability at antenatal care consultations or pre-pregnancy consultations. Or due to the complexity of vulnerability – not knowing where to start, i.e. having to discuss too many different health issues, making GPs feeling overwhelmed and finding it difficult to make the decision on how to prioritize the level of information given. Decision processes may relate to the GPs choice of listening to their gut feelings in the decision of whether the woman is vulnerable or not.
COM-B: psychological CAPABILIY	**Behavioral regulation (14)**<br>• Self-monitoring *(keeping records of behavior in connection with efforts to change or regulate, the ability to modify one's behavior in response to a situation)*<br>• Action planning	• Statements where GPs wants audit/evaluation/feedback on their assessment of vulnerability from cross-sectoral collaborators<br>• Statements about prompts or processes used to make the assessment process sustainable<br>• Statements about efforts to ensure assessment of vulnerability

	• Project management	In the context of this study behavioral regulation may relate primarily to the need for use of prompts relating to the assessment such as having specific notifications in women's files notes that relate to vulnerability determinants
COM-B: reflective MOTIVATION	**Social and professional role and identity (self-standards) (3)**<br>• Professional identity *(The characteristics by which an individual is recognised relating to, connected with or befitting a particular profession)*<br>• Professional role *(The behaviour considered appropriate for a particular kind of work or social position)*<br>• Social identity *(The set of behavioural or personal characteristics by which an individual is recognizable [and portrays] as a member of a social group)*<br>• Identity *(An individual's sense of self defined by a) a set of physical and psychological characteristics that is not wholly shared with any other person and b) a range of social and interpersonal affiliations (e.g., ethnicity) and social roles)*<br>• Professional boundaries *(The bounds or limits relating to, or connected with a profession or calling)*<br>• Professional confidence *(An individual's belief in his or her repertoire of skills, and ability especially as it is applied to a task or set of tasks)*<br>• Group identity *(The set of behavioural or personal characteristics by which an*	• Statements relating to how GPs' sees themselves<br>• Statements relating to the extent they view assessment of vulnerable pregnant women as a characteristic/meaningful task representative of their professional role<br>• Statements relating to the extent their personal identity influences assessment of vulnerable pregnant women<br>○ Part of our role/job/responsibility to do the behavior<br>○ My personal identity impacts on how I assess for vulnerability in pregnancy (being a mother/father)<br>In the context of this study, professional role may relate to the extent that GPs' feels that providing risk assessment and collaborating about vulnerable pregnant women is part of their professional role, and the roles of other HCP groups. May relate to professionals' gender or status as mother/father and the impact it has on vulnerability support. Personal identity may relate to the GPs gender or status as being a parent and the impact this has on assessing and addressing vulnerability

	individual is recognizable [and portrays] as a member of a group)<br>• Leadership *(The processes involved in leading others, including organising, directing, coordinating and motivating their efforts toward achievement of certain group or organisation goals)*<br>• Organizational commitment (*emotional or moral element, prudent element – a dedication to an organization wishing to remain part of it)*	
COM-B: reflective MOTIVATION	**Beliefs about capability (self-efficacy) (4)**<br>• Self-confidence *(trust in one's own abilities, capabilities, and judgement)*<br>• Perceived competence (*belief in his/her ability to learn and execute skills)*<br>• Perceived behavioral control *(perception of the ease or difficulty of performing the behavior of interest)*<br>• Self-esteem (*the degree to which the qualities and characteristics contained in one's self-*concept *are perceived to be positive)*<br>• Empowerment (*the promotion of the skills, knowledge, and confidence necessary to take great control of one's life; as in certain educational or social schemes; the delegation of increased decision-making powers to individuals or groups in a society or organization)*<br>• Professional confidence (*an individual's belief in his/her repertoire of skills, and ability – especially as it is applicated to a task or set of tasks)*<br>• Control of behavior and material and social environment	• Evaluative statements about GPs' confidence and judgement about their competence and control in their ability or inability to perform the risk assessment and collaborate in antenatal care for vulnerable pregnant women.<br>○ I feel that I have/don't have control in assessing vulnerable pregnant women<br>• Statements relating to expectations of carrying out the assessment due to beliefs of own competencies.<br>○ I know that assessing/addressing…. won't be successful because I'm/I'm not very efficient at doing this task<br>In the context of this study, beliefs about capability relates to the GPs making evaluative judgements on their ability to assess or address vulnerability, for example their confidence in being able to have an overview of the antenatal care for vulnerable pregnant women- or to sensitively address and discuss women's vulnerability and subsequent risks in pregnancy.

COM-B: reflective MOTIVATION	**Optimism (5)**<br>• Optimism: *the confidence that things will happen for the best or that desired goals will be attained)*<br>• Pessimism (*the attitude that things will go wrong and that peoples wishes or aims are unlikely to be fulfilled)*	• Optimism or pessimism –confidence that the problem can be solved.<br><u>In the context of this study,</u> this would include expressing optimism/pessimism of the effects of addressing vulnerability, referring for social-obstetric specialized care, and reporting to municipalities based directly upon their believed competence in these behaviors.
COM-B: reflective MOTIVATION	**Beliefs about consequences (anticipated outcomes/attitude) (6)**<br>• Beliefs *(The thing believed; the proposition or set of propositions held true)*<br>• Outcome expectancies *(cognitive, emotional, behavioral and affective outcomes that are assumed to be associated with future or intended behaviors. These assumed outcomes can either promote or inhibit future behaviors)*<br>• Characteristics of outcome *expectancies (characteristics of the cognitive, emotional and behavioral outcomes that individuals believe are associated with future or intended behaviors and that are believed to either promote or inhibit these behaviors. These include whether they are sanctions/rewards, probable/improbable, perceived risk or threats)*<br>• Anticipated regret (*a sense of the potential negative consequences of a decision that influences the choice made)*<br>• Consequents (*an outcome of behavior in a given situation)*	• Statements relating to GPs beliefs about the outcome/consequences of addressing/not addressing vulnerability –<br>• Statements can include the positive or negative consequences of assessing/not assessing vulnerability in pregnancy<br>• Statements can include consequences of assessing/not assessing vulnerability in pregnancy – or collaborating/not collaborating about vulnerable pregnant women on themselves or their patient<br>○ If I do/don't do …. X, y, z will happen<br>○ Assessing vulnerability will have a beneficial /adverse impact on me/my patient<br>○ Collaborating/not collaborating, positive or negative consequences of collaborating<br><u>In the context of this study,</u> beliefs about consequences could relate to GPs beliefs that addressing vulnerability- or engaging in the cross-sectoral collaboration about vulnerable pregnant women will result in damage of the doctor-patient relationship/alliance, etc. Also, willingness to perform the risk assessment based on expectations of outcomes (ex it is perceived pointless/too late to reduce risks etc.
COM-B: Reflective MOTIVATION	**Intentions (8)**<br>A conscious decision to perform a behavior in a certain way<br>• Stability of intentions: (ability of one's resolve to remain despite disturbing influences)<br>• Stages of change model	• Things I want to do<br>• Statements relating to the extent that GPs planning to assess vulnerability, or to engage in the cross-sectoral collaboration in antenatal care of vulnerable pregnant women.

	• Trans theoretical model and stages of change: (a five-stage theory to explain changes in health behavior. It suggests that change takes time, that different interventions are effective at different stages, and that there are multiple outcomes occurring across stages)<br>• Intrinsic motivation<br>• Commitments	In the context of this study, intentions may be GPs' stating how they aims to always discuss vulnerability determinants when seeing a pregnant woman. Conversely weak intentions may be a lack of intention to assess for indicators of vulnerability (e.g. I don't always make a point of addressing….)
COM-B: Reflective MOTIVATION	**Goals (9)**<br>Mental representations of outcomes or end states that an individual wants to achieve<br>• Goals: desired state of affairs of a system or person, may be proximal (closer) or distal (further away)<br>• Goal priority<br>• Goal target setting specific time-based behavior targets that are measurable, achievable, and realistic	• Things I want to achieve<br>• Statements relating to GPs goals/aims/desired result of doing the behavior (ex. reporting to authorities),<br>• Statements relating to how prioritizing goals influences whether to do the behavior<br>○ competing priorities mean I don't assess vulnerability<br>○ I prioritize other behaviours which are more important<br>In the context of this study, GPs goals may relate to wanting to support vulnerable pregnant women during their pregnancy to get the right supportive care, their family's health, and pregnancy outcomes, to reduce risks. Goal priorities may relate to the competing topics to cover during antenatal appointments and how important antenatal care is perceived in relation to other priorities of primary care.
COM-B: automatic MOTIVATION	**Reinforcement (7)**<br>• Rewards *(proximal / distal, valued / not valued, probable /improbable) (Return or recompense made to, or received by a person contingent on some performance)*<br>• Incentives (*an external condition that enhances or serves as a motive for behavior*)<br>• Punishment (*an unwanted or undesired event imposed as a penalty on a wrongdoer*)<br>• Consequents (*an outcome of behavior in a given situation*)<br>• Reinforcement (Increasing the probability of a response by arranging a dependent relationship or	• Statements relating to assessing/addressing/collaborating being directly contingents on receiving rewards or punishments<br>○ Otherwise x, y, z will /will not happen<br>○ Getting thanked for encourages me to do….<br>○ I will get in trouble/be reported if I don't…<br><br>In the context of this study, reinforcement could relate to doing risk assessment of vulnerability because not assessing for vulnerability/collaborating about vulnerable pregnant women is linked with punishments such as litigation/complaints. Reinforcements could also be related to the incentives that motivates for assessing vulnerability.

	contingency between the response and a given stimulus)<br>• Contingencies *(A conditional probabilistic relation between two events. Contingencies may be arranged via dependencies or they may emerge by accident)*<br>• Sanctions (*A punishment or other coercive measure, usually administered by a recognised authority, that is used to penalise and deter inappropriate or unauthorised actions*	
COM-B: Automatic MOTIVATION	**Emotion (13)**<br>• Fear *(An intense emotion aroused by the detection of imminent threat, involving an immediate alarm reaction that mobilises the organism by triggering a set of physiological changes)*<br>• Anxiety *(A mood state characterised by apprehension and somatic symptoms of tension in which an individual anticipates impending danger, catastrophe, or misfortune)*<br>• Affect *(An experience or feeling of emotion, ranging from suffering to elation, from the simplest to the most complex sensations of feelings, and from the most normal to the most pathological emotional reactions)*<br>• Stress *(A state of physiological or psychological response to internal or external stressors)*<br>• Depression *(A mental state that presents with depressed mood, loss of interest or pleasure, feelings of guilt or low self-worth, disturbed sleep or appetite, low energy, and poor concentration)*<br>• Positive / negative affect *(The internal feeling/state that occurs when a goal has/has not been attained, a source of threat has/has not been avoided, or the individual is/is not satisfied with the present state of affairs)*	• An expression of GP own personal emotional reaction,<br>• Expression how this positively/negatively impact on them assessing vulnerability in pregnancy, in this context the emotional response of the GP in relation to addressing vulnerability and collaborate/inform local authorities => fear/anxiety, affect, stress, depression, positive/negative affect, burn-out.<br>In the context of this study, emotion relates to the emotional responses of the GPs, and not the emotional responses of the patients in relation to assessing vulnerability<br>Ex when bur-out impedes the reaction of cress-sectoral collaboration, or cross-sectoral collaboration gives burn-out

<table>
<tr><td></td><td>Burn-out (Physical, emotional, or mental exhaustion, especially in one's job or career, accompanied by decreased motivation, lowered performance and negative attitudes towards oneself and others)
Cognitive overload / tiredness (The situation in which the demands placed on a person by mental work are greater than a person's mental abilities)</td><td></td></tr>
<tr><td>COM-B:
physical
OPPORTUNITY</td><td>Environmental context and resources (11)
Environmental stressors (external factors in the environment that cause stress)
Resources/material resources (availability and management- human resources used in enacting a behavior)
Organizational culture/climate (a distinctive pattern of thought and behavior shared by members of the same organization and reflected in their language, values, attitudes, beliefs and customs)
Critical incidents (occurrences that one judges to be distinctive, prominent or otherwise significant)
Person x environment interaction (interplay between the individual and their surroundings)
Knowledge of task environment (knowledge of the social and material context in which a task is undertaken)</td><td>Statements describing the presence/absence of tools/resources/equipment/services/organizational structures which facilitates/limits the GPs in assessing and addressing vulnerability and their cross-sectoral collaboration in antenatal care for vulnerable pregnant women.
Wished tools/resources/services/changes in the organizational structure to facilitate performing the risk assessment
Services/resources to improve the assessment of vulnerability
The environment/organizational culture has an impact on the assessment of vulnerability
<u>In the context of this study, e</u>xamples on the environmental contest and resources could be the availability of support services, patient- or HCP information, service-level pathways of care for vulnerable pregnant women, time, staffing levels, whether organization culture prioritizes/provides resources for assessment of vulnerability etc. (NOTE: in relation to having to priorities behaviours due to time restrictions then time would be coded as goals)</td></tr>
<tr><td>COM-B:
social
OPPORTUNITY</td><td>Social influences (12) (those interpersonal processes that can cause individuals to change their thoughts, feelings or behaviors)
Social pressure (the exertion of influence on a person or group by another person or group)</td><td>Statements expressing the influence of others (GPs, HCPs, patients and families) on doing/not doing the behavior (social support, group norms etc.)
I do/don't do assess/address vulnerability in pregnancy because others support/advocate/disapprove/dictate/demand it</td></tr>
</table>

	• Social norms (*socially determined consensual standards)*<br>• Group conformity *(the act of consciously maintaining a certain degree of similarity to those in your general social circles)*<br>• Social comparisons (*the process by which people evaluate their attitudes, abilities or performance relative to others*)<br>• Group norms *(any behavior, belief, attitude or emotional reaction held to be correct or acceptable by a given group in society)*<br>• Social support<br>• Power<br>• Intergroup conflict<br>• Alienation (deep sense of dissatisfaction with one's personal experiences that can be a source of lack of trust in one's social or physical environment or in oneself)<br>• Group identity (*set of behavioral or personal characteristic by which an individual is recognizable and portrays as a member of a group)*<br>• Modelling<br>• Organizational culture/climate *(a distinctive pattern of thought and behavior shared by members of the same organization and reflected in their language, values, attitudes, beliefs and customs)*	In the context of this study, others may include individuals or groups of peers, cross-sectoral collaborators (hospital specialist doctors, midwifes, nurses, health-visitors, social workers) or authoritative organizations etc. NOTE: not the interpersonal behavior addressing vulnerability = skills)
